# Cottonseed oil alleviates ischemic stroke injury by inhibiting the inflammatory activation of microglia and astrocyte

**DOI:** 10.1186/s12974-020-01946-7

**Published:** 2020-09-11

**Authors:** Min Liu, Zhipeng Xu, Long Wang, Lixia Zhang, Yi Liu, Jiangbei Cao, Qiang Fu, Yanhong Liu, Hao Li, Jingsheng Lou, Wugang Hou, Weidong Mi, Yulong Ma

**Affiliations:** 1 Anesthesia and Operation Center, The First Medical Center of Chinese PLA General Hospital, Beijing, 100853 China; 2 Department of Burn and Plastic Surgery, The Fourth Medical Center of Chinese PLA General Hospital, Beijing, 100048 China; 3 Department of Anesthesiology and Perioperative Medicine, Xijing Hospital, Air Force Military Medical University, Xi’an, 710032 China

**Keywords:** Cottonseed oil (CSO), Ischemic stroke, Neuroinflammation, Microglia, Astrocyte, Neuroprotection

## Abstract

**Background:**

Ischemic stroke is the second leading cause of death globally. The narrow time window for administering effective thrombolytic therapy motivates the search for alternative prevention strategies. Microglia and astrocyte activation-mediated inflammation play a pivotal role in ischemic stroke injury. Cottonseed oil (CSO) has been shown to exert anti-inflammatory effects against peripheral tissue injury, although CSO is mostly used as a solvent for lipid-soluble drugs. However, the role of CSO in neuroprotection against stroke has not been previously reported.

**Methods:**

We treated adult male rats with CSO (1.3 ml/kg, subcutaneous injection, once every other day for 3 weeks) and then constructed a middle cerebral artery occlusion (MCAO) model followed by 24 h of reperfusion. Then, we measured the neurological scores, infarction volume, neuronal injury, and brain edema; we also measured the levels of pro-inflammatory cytokines (IL-1β, IL-6, TNF-α), degree of microglial and astrocytic activation, protein expression levels of Toll-like receptor 4 (TLR4), nuclear factor kappa B (NF-κB), C3d and S100A10, and the presence of A1 type astrocytes and A2 type astrocytes.

**Results:**

We found that CSO treatment significantly improved the neurological deficit, reduced infarction volume, and alleviated neuronal injuries, blood–brain barrier (BBB) disruption, and brain edema. Additionally, CSO treatment significantly reduced microglial and astrocytic activation, inhibited TLR4 and NF-κB protein expression, and reduced the release of IL-1β, IL-6, and TNF-α. Finally, CSO treatment significantly decreased the number of C3d/glial fibrillary acidic protein (GFAP)-positive cells and C3d protein expression, and increased the number of S100A10/GFAP-positive cells and S100A10 protein expression.

**Conclusion:**

Our results first found that CSO treatment alleviated ischemic stroke injury by reducing microglial and astrocytic activation and inflammation, which was related to the inhibition of TLR4/NF-κB pathway and the reduction of A1 phenotype neurotoxic astrocyte activation, suggesting that CSO could be a new strategy in the prevention of ischemic stroke.

## Introduction

Stroke is an important contributor to global mortality and disability burden, with 10.3 million new stroke cases per year [1]. Currently, thrombolytic therapy is still the most effective treatment for ischemic stroke, but only a small number of patients receive effective thrombolytic therapy within its narrow therapy time window (4 to 6 h) after stroke onset [2, 3]. Therefore, it is critical to develop effective prevention strategies.

Cottonseed oil (CSO), a valuable type of vegetable oil, is made up of linoleic acid (52–65%), oleic acid (25–36%), and palmitic acid (6–8%) [4]. In previous studies, CSO has primarily been used as the solvent for lipid-soluble drugs such as estrogen and ketorolac [5, 6]. However, several studies have found that CSO exerts protective effects against peripheral tissue injury such as gastrointestinal disease, principally by reducing inflammatory response [7, 8]. Additionally, while high lipid intake has been suggested as one of the main factors leading to atherosclerosis, this is not the case for diets containing CSO as a source of fat [9]. However, the specific effects and underlying mechanisms by which CSO treatment may alleviate ischemic stroke injury have not been systematically studied.

A number of both of clinical trials and basic research studies have shown that microglial and astrocytic activation mediate the inflammation responses which underpin ischemic stroke injury [10–12]. For example, inhibiting microglia and astrocyte activation alleviates ischemic stroke injury by reducing inflammation responses in the central nervous system (CNS) [13, 14]. Toll-like receptor 4 (TLR4) and nuclear factor kappa B (NF-κB) have been identified as the key molecules leading to microglia and astrocyte activation following ischemic stroke [15, 16]. Recent studies indicate that ischemic stroke induces two different polarized states of reactive astrocytes, termed neurotoxic A1 type and neuroprotective A2 type [17]. A1 type astrocytes, which are induced by the IL-1α, TNF-α, and C1q secreted by activated microglia, release the neurotoxic complement C3d directly, leading to neuron death. Inhibiting the activation of A1 type astrocytes leads to significant neuroprotective effects [17, 18]. These findings indicate the importance of modulating the activation of microglia and astrocytes for ischemic stroke treatment. However, while previous research suggests that CSO reduces inflammatory responses, it is not known whether CSO affects the inflammation caused by microglial and astrocytic activation following ischemic stroke injury.

In the current study, we treated male rats with CSO and then performed middle cerebral artery occlusion (MCAO). We measured neurological scores, infarction volume, neuronal injury, and brain edema; we also measured levels of pro-inflammatory cytokines (IL-1β, IL-6, TNF-α), degree of microglial and astrocytic activation, protein expression levels of TLR4, NF-κB, C3d, and S100A10, and the presence of A1 type astrocytes and A2 type astrocytes.

## Methods and materials

### Animals

One hundred and eighty four male adult SD rats weighing 280–350 g were obtained from the Laboratory Animal Center of Chinese PLA General Hospital. These rats were randomly assigned to four groups according to whether they received CSO treatment and/or underwent middle cerebral artery occlusion (MCAO) and reperfusion injury (*n* = 46 per group): sham group without any treatment (Sham-Con), sham plus CSO treatment group (Sham-CSO), MCAO-R injury group without treatment (MCAO-Con), MCAO-R injury plus CSO treatment group (MCAO-CSO). Rats were housed according to the standardized conditions. All experimental procedures strictly complied with relevant ethical regulations and were approved by the Ethics Committee for Animal Experimentation of the Chinese PLA General Hospital.

### CSO treatment

The rats in Sham-CSO and MCAO-CSO groups received subcutaneous injection of 1.3 ml/kg cotton seed oil (CSO) (J&K, China) every other day for 3 weeks. Researchers performing animal experiments were blinded to the treatment. The concentrations selected in this study were based on the concentrations as the solvent administered in previous study [5].

### Middle cerebral artery occlusion and reperfusion (MCAO-R)

The rats in MCAO-Con and MCAO-CSO groups received MCAO-R injury as previously described [15]. Rats were anesthetized with 10% chloral hydrate (3 ml/kg), and the body temperatures were continuously monitored and maintained at 36.5–37.5 °C with a thermostatic blanket. Following 1 h of transient occlusion, cerebral blood flow was restored by removing the suture for 24 h. Regional cerebral blood flow was monitored by laser-Doppler flowmetry (Supplementary Fig. 1). Only the rats whose mean cortical cerebral blood flow decreased to 20% of the pre-ischemic value and recovered to 80% of the baseline after reperfusion were used for data analysis.

### Bederson test

Bederson test was performed 24 h after reperfusion to evaluate neurobehavioral deficits as previously described (*n* = 10) [19]. The neurological evaluation was scored using a 5-point scale: 0, no neurological deficit; 1, failure to extend left forepaw fully; 2, circling to the left; 3, inability to bear weight on the left; 4, no spontaneous walking with depressed level of consciousness.

### Assessment of infarct volume

After neurological score, infarct volume was assessed via 2,3,5-triphenyltetrazolium chloride (TTC) staining as previously described (*n* = 10) [20]. Then, the brain slices were photographed, and infarct volume (the unstained areas) was determined using image analysis software (Adobe Photoshop CS6). Corrections were made for swelling, and relative infarct size was determined based on the following equation: relative infarct size = (contralateral area − ipsilateral non-infarct area)/contralateral area.

### HE staining

Hematoxylin and eosin (HE) staining was used to detect the pathological changes in the ischemic penumbra 24 h after reperfusion (*n* = 5). Briefly, the rats were anesthetized as above, and the brains were fixed via transcardial perfusion with 0.9% cold heparinized saline and 4% paraformaldehyde, paraffin-embedded, and sectioned at a thickness of 4 μm for HE staining, Nissl staining, and TUNEL staining. Then, the sections were stained with HE, and the number of HE-positive cells in the ischemic penumbra was counted in 5 different fields for each section in a blinded manner using a light microscope (BX51; Olympus, Tokyo, Japan).

### Nissl staining

Nissl staining was applied to observe neuronal morphologic changes in the ischemic penumbra 24 h after reperfusion (*n* = 5). The experimental steps were strictly performed according to the manufacturer’s manual of the Nissl staining kit (#G1430, Solarbio, China). The total number of Nissl-positive neurons in the penumbra was counted in 5 different fields of view for each section by an observer blinded to the treatment group manner via a light microscopy (BX51; Olympus, Tokyo, Japan).

### TUNEL staining

TUNEL Staining was performed to observe cell apoptosis in the ischemic penumbra 24 h after reperfusion (*n* = 5). TUNEL staining was administered according to the manufacturer’s instructions of In Situ Cell Death Detection kit (Roche Diagnostics, Germany). TUNEL-positive neurons/DAPI were regarded as an apoptosis index. The number of TUNEL-positive cells in the ischemic penumbra was counted in 5 different fields of view for each section in a blind manner via a light microscopy (BX51; Olympus, Tokyo, Japan).

### Immunofluorescence staining

Immunofluorescence staining was performed on frozen coronal sections of rat brains to quantify cells in the ischemia penumbra 24 h after reperfusion (*n* = 5). After post-fixation and concentration gradient dehydration, the brains were cut into 10-μm-thick sections using a Leica CM1900 frozen slicer. The brain sections were washed three times with PBS and then incubated overnight at 4 °C in a humidified atmosphere with primary antibodies. The following primary antibodies were used: mouse anti-NeuN (1:200; Millipore, USA), mouse anti-GFAP (1:200; Cell Signaling Technology, USA), rabbit anti-Iba1 (1:500; Wako, Japan), rabbit anti-TLR4 antibody (1:1000, Cell Signaling Technology), mouse anti-phospho-NF-κB p65 (Ser536) antibody (1:1000, Abcam, USA), rabbit anti-TNF-α (1:100; Cell Signaling Technology), mouse anti-TNF-α (1:100; Abcam), goat anti-C3d (1:50; RD Systems, USA), rabbit anti-S100A10 (1:500; Invitrogen, USA). Then, the samples were incubated with mixtures of Alexa-488 (green, Invitrogen) or Alexa-594 (red, Invitrogen) and Alexa-647 (red, Invitrogen)-conjugated donkey anti-goat or anti-rabbit and donkey anti-mouse secondary antibodies for 2 h in the dark at room temperature. The sections were mounted with 50% glycerol and examined under a fluorescence microscope. The total number of NeuN-positive neurons, GFAP-positive astrocytes, Iba1-positive microglia, C3d/GFAP-positive cells, and S100A10/GFAP-positive cells respectively in the ischemic penumbra were counted in 5 different fields of view for each section by an observer blinded to the treatment group manner via light microscopy (BX51; Olympus, Tokyo, Japan).

### Brain water content

Brain water content was measured by the wet/dry weight method [21]. Rats (*n* = 6) were anesthetized, and the brains were removed quickly at 24 h after reperfusion. A coronal brain slice (about 5-mm thick) 3 mm from the frontal pole was cut and divided into the ischemic and non-ischemic hemispheres. Ischemic hemisphere samples were weighed wet and then dried at 100 °C in a vacuum oven for 48 h and reweighed. Brain water content (%) was calculated as ((wet weight − dry weight)/wet weight) × 100%.

### Determination of the blood–brain barrier (BBB) permeability

Immediately after reperfusion, 4 ml/kg of 2% Evans blue (Sigma Aldrich) was injected into the right jugular vein (*n* = 6). The animals were euthanatized 24 h after reperfusion. For Evans blue extravasation assay, coronal sections were cut into 2-mm slices and were photographed. The blue-stained area was defined as BBB permeable. For quantitative measurements, the ischemic hemispheres were homogenized in N, N-dimethylformamide (10 ml/kg, Sigma Aldrich), incubated for 18 h at 55 °C, and centrifuged at 3000 rpm for 10 min [22]. The supernatants were analyzed at 620 nm by spectrophotometry. The EB content was calculated from its standard curve as a measure of the BBB permeability change.

### Transmission electron microscopy

Rats (*n* = 3) were anesthetized and intracardially perfused with 0.1 M phosphate-buffered solution (PBS) containing 0.1% glutaraldehyde. The ischemia penumbras of rats were dissected and postfixed overnight at 4 °C using 2.5% glutaraldehyde. Tissues were cut along the coronal plane at a thickness of 50 μm. Samples were processed for TEM observation as previously described [23].

### Detection of proinflammatory cytokines

The ischemia penumbra tissues were homogenized in PBS 24 h after reperfusion; then, the tissue fluids of the ischemia penumbra were collected (*n* = 5). Concentrations of interleukin-1β (IL-1β), interleukin-6 (IL-6), and tumor necrosis factor α (TNF-α) were examined using an ELISA kit following the manufacturer’s instructions (Biosource, Invitrogen, USA). Absorbance was measured at 450 nm using a microplate reader (Infinite M2000, TECAN, Switzerland).

### Western blot analysis

Fifty milligrams of ischemic penumbra tissues (*n* = 6) was used for Western blot analysis as previously described [15]. The primary antibodies include the following: mouse anti-VE-cadherin (1:1000, Cell Signaling Technology), rabbit anti-AQP4 (1:1000, Proteintech, USA), rabbit anti-Claudin-1 (1:1000, Abcam, USA), mouse anti-GFAP (1:1000, Cell Signaling Technology), rabbit anti-Iba1 antibody (1:500, Wako), goat anti-C3d antibody (1:1000, RD Systems), rabbit anti-S100A10 (1:1000; Invitrogen), rabbit anti-TLR4 antibody (1:1000, Cell Signaling Technology), mouse anti-phospho-NF-κB p65 (Ser536) antibody (1:1000, Abcam), mouse anti-NF-κB p65 antibody (1:1000, Abcam), mouse anti-GAPDH (1:1000, Cell Signaling Technology). The membranes were then incubated with the corresponding HRP-conjugated secondary antibody for 2 h. Protein bands were visualized using the LI-COR Odyssey System (LI-COR Biotechnology, USA).

### Statistical analysis

All data were analyzed by an observer who was blind to the experimental protocol. Statistical calculations were performed with the GraphPad Prism software, version 8.0. All results, except for neurological deficit scores, were expressed as mean ± standard deviation (SD), applying a two-way ANOVA with Tukey’s post hoc test to determine significant differences between the experimental groups. The neurological deficit scores were presented as median with interquartile range and were analyzed using two-tailed Mann-Whitney *U* tests. *P* values < 0.05 were considered statistically significant.

## Results

### CSO treatment significantly attenuated cerebral ischemic injury

**Fig. 1:**
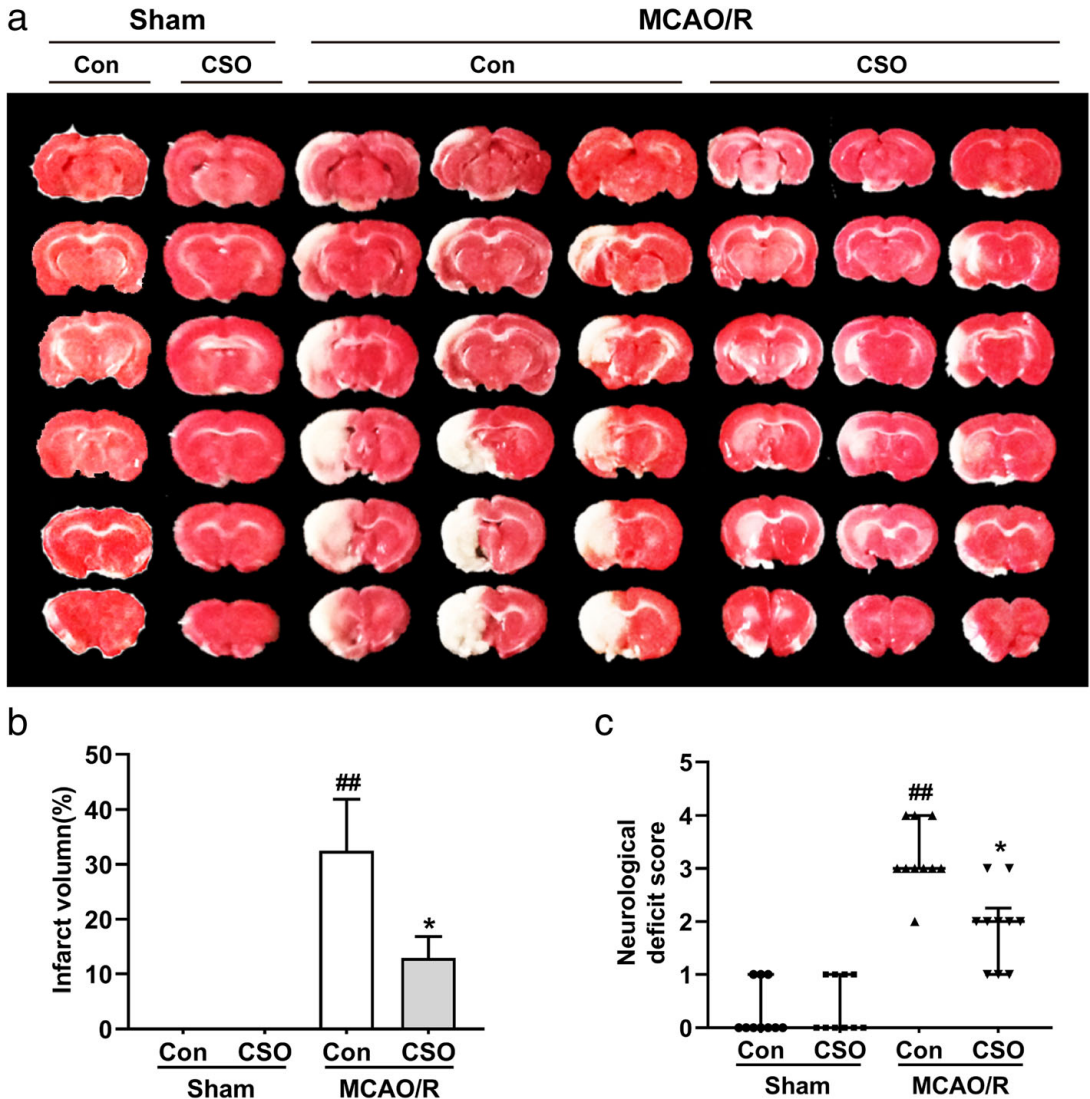
CSO treatment reduced cerebral ischemic injury. **a** Representative photographs of brain slices showing the infarct volume assessed 24 h after reperfusion. **b** Statistical analysis of infarction volume in different groups. ^##^*p* < 0.01 vs. Sham-Con group, ^*^p < 0.05 vs. MCAO-Con group. *n* = 10 per group. **c** Neurological deficit score evaluated 24 h after reperfusion. ^##^*p* < 0.01 vs. Sham-Con group, ^*^p < 0.05 vs. MCAO-Con group. *n* = 10 per group

First, we used neurological scores and TTC staining to measure brain injury in the four groups. As shown in Fig. 1a and b, the infarct volume in the MCAO-Con group was 32.5% ± 9.4% (^##^*p* < 0.01 vs. Sham-Con group). CSO treatment significantly decreased the infarct volume to 12.9% ± 3.9% (^*^p < 0.05 vs. MCAO-Con group). Additionally, as shown in Fig. 1c, there was no significant difference in the neurological scores of the rats between the Sham-Con and Sham-CSO groups. Compared with the Sham-Con group, the MCAO-Con group had significantly higher neurological scores, indicating a worse neurological condition (^##^*p* < 0.01). Interestingly, the neurological scores of the rats in the MCAO-CSO group was dramatically reduced compared with that of the MCAO-Con group (^*^p < 0.05).

### CSO treatment significantly alleviated neuronal injury in the ischemia penumbra

**Fig. 2:**
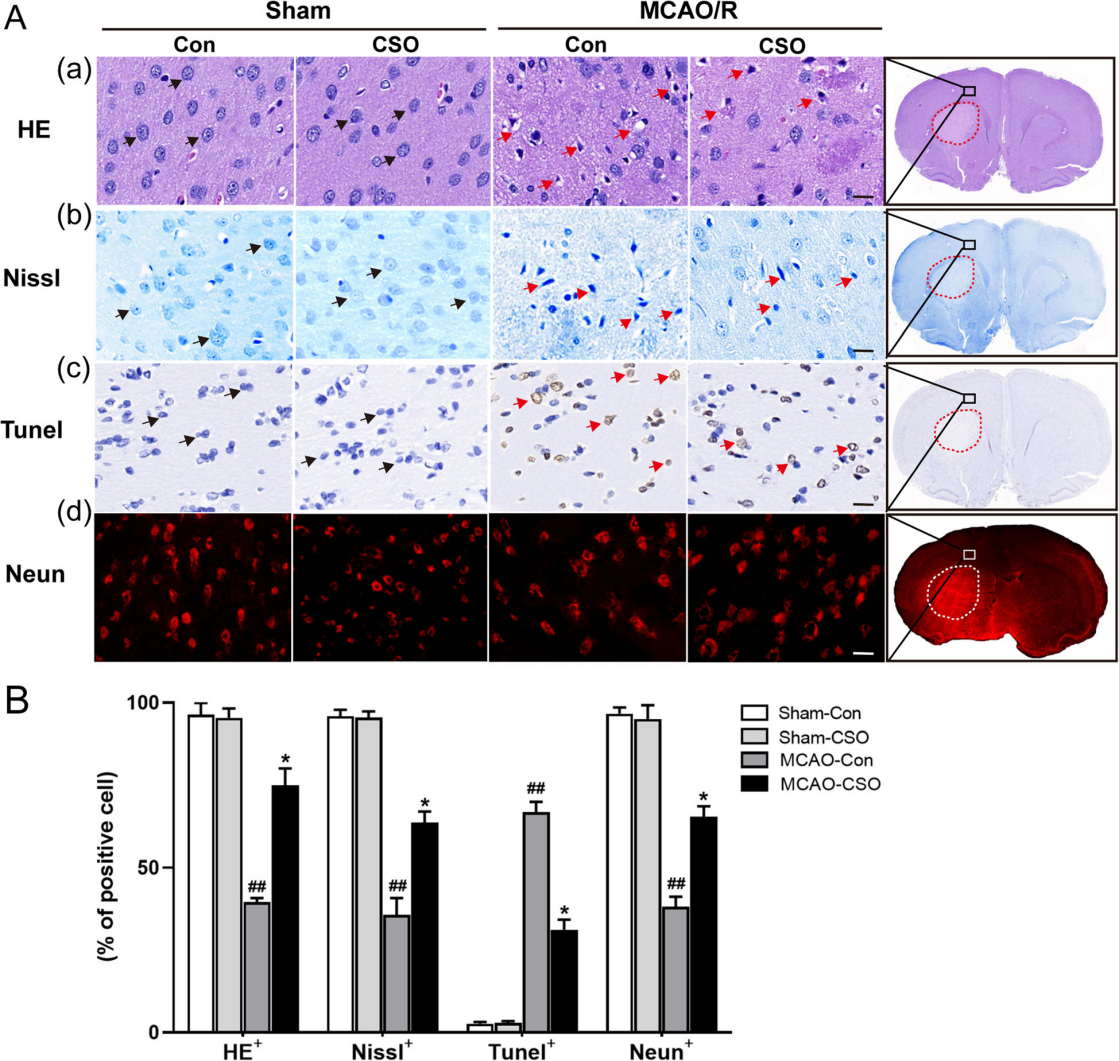
CSO treatment alleviated neuronal injury in the ischemic penumbra. **A(a)** HE staining showing cell morphologic changes in the ischemic penumbra 24 h after reperfusion. Scale bars = 10 μm. Black arrows represent the cell outline was clear and structure was compact. Red arrows represent the cells were arranged sparsely, cell outline was fuzzy, and structure was disorder. **A(b)** Nissl staining showing morphological neuronal changes in the ischemic penumbra 24 h after reperfusion. Scale bars = 10 μm. Black arrows represent the intact neurons with flush cell bodies. Red arrows represent the injured neurons with shrunken cell bodies accompanied by shrunken and pyknotic nuclei. **A(c)** TUNEL staining showing the neuronal apoptosis in the ischemic penumbra 24 h after reperfusion. Scale bars = 10 μm. Black arrows represent the intact cells with flush cell bodies. Red arrows represent apoptotic nerve cells with shrunken and pyknotic nuclei. **A(d)** NeuN staining showing the survival of neurons in the ischemic penumbra 24 h after reperfusion. Scale bars = 20 μm. **B** The percentage of complete cells (HE^+^), intact neurons (Nissl^+^), neuronal apoptosis (Tunel^+^), and living neurons (Neun^+^) in the ischemic penumbra. ^##^*p* < 0.01 vs. Sham-Con group, ^*^p < 0.05 vs. MCAO-Con group. *n* = 5 per group

HE, Nissl, TUNEL, and NeuN staining were performed to assess the neuronal damage and survival in the ischemic penumbra following MCAO-R injury (Fig. 2). As shown in Fig. 2A (a) and 2B, cells that were positive following HE staining (HE^+^) had a clear outline, compact structure, and intact nucleolus. The proportion of HE^+^ cells was 96.4% ± 3.5% in the Sham-Con group and 95.4% ± 2.8% in the Sham-CSO group, with no significant difference between the groups. The proportion of HE^+^ cells was 39.6% ± 1.2% in the MCAO-Con group (^##^*p* < 0.01 vs. Sham-Con group), and CSO treatment significantly increased the proportion of HE^+^ cells to 75.1% ± 5.0% (^*^p < 0.05 vs. MCAO-Con group).

As shown in Fig. 2A (b) and B, cells that were positive following Nissl staining (Nissl^+^) had intact neurons with flush cell bodies, while injured neurons had shrunken cell bodies accompanied by shrunken and pyknotic nuclei. The proportion of Nissl^+^ cells was 95.9% ± 1.9% in the Sham-Con group and 95.5% ± 1.8% in the Sham-CSO group, with no significant difference between the groups. The proportion of Nissl^+^ cells was 35.6% ± 5.2% in the MCAO-Con group (^##^*p* < 0.01 vs. Sham-Con group), and CSO treatment significantly increased the proportion of Nissl^+^ cells to 63.6% ± 3.3% (^*^p < 0.05 vs. MCAO-Con group).

As shown in Fig. 2A (c) and B, cells that were positive following TUNEL staining (Tunel^+^) represented apoptotic nerve cells. The proportion of Tunel^+^ cells was 2.6% ± 0.5% in the Sham-Con group and 2.9% ± 0.6% in the Sham-CSO group, with no significant difference between the groups. The proportion of Tunel^+^ cells was 66.8% ± 3.1% in the MCAO-Con group (^##^*p* < 0.01 vs. Sham-Con group), and CSO treatment significantly decreased the proportion of Tunel^+^ cells to 31.2% ± 3.1% (^*^p < 0.05 vs. MCAO-Con group).

Finally, as shown in Fig. 2A (d) and B, cells that were positive following NeuN staining (Neun^+^) reflect surviving neurons. The proportion of Neun^+^ cells was 96.7% ± 1.9% in the Sham-Con group and 95.0% ± 4.2% in the Sham-CSO group, with no significant difference between the groups. The proportion of Neun^+^ cells was 38.2% ± 3.1% in the MCAO-Con group (^##^*p* < 0.01 vs. Sham-Con group), and CSO treatment significantly increased the proportion of Neun^+^ cells to 65.5% ± 3.2% (^*^p < 0.05 vs. MCAO-Con group).

### CSO treatment alleviated brain edema induced by ischemic stroke

**Fig. 3:**
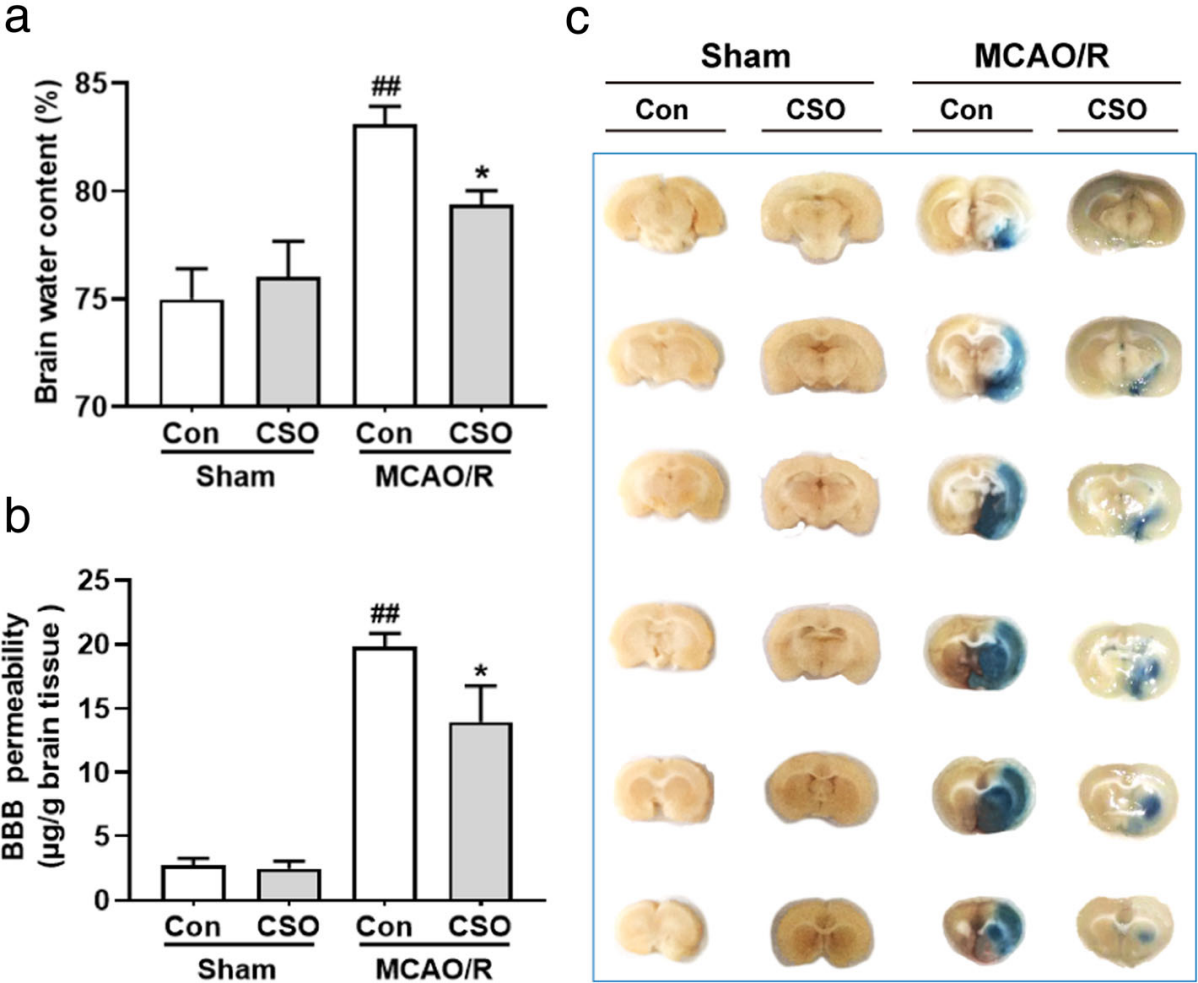
CSO treatment rescued brain edema after ischemic stroke. **a** Quantification of brain water content in ischemic hemisphere isolated from different groups. ^##^*p* < 0.001 vs. Sham-Con group, ^*^p < 0.05 vs. MCAO-Con group. *n* = 6 per group. **b** Quantitative assay of Evans blue leakage in rats from different groups 24 h after reperfusion. ^##^*p* < 0.001 vs. Sham-Con group, ^*^p < 0.05 vs. MCAO-Con group. *n* = 6 per group. **c** Representative photographs of Evans blue extravasation in the brains and coronal sections in rats from different groups 24 h after reperfusion

In order to determine the extent of brain edema 24 h after reperfusion, we measured brain water content and blood-brain barrier (BBB) permeability. As shown in Fig. 3a, the brain water content was 75.0% ± 1.4% in the Sham-Con group and 76.0% ± 1.7% in the Sham-CSO group, with no significant difference between the groups. The brain water content was 83.1% ± 0.8% in the MCAO-Con group (^##^*p* < 0.01 vs. Sham-Con group), and CSO treatment significantly decreased the brain water content to 79.4% ± 0.6% (^*^p < 0.05 vs. MCAO-Con group).

As shown in Fig. 3B and C, the content of Evans blue was 2.7 μg/g ± 0.5 μg/g in the Sham-Con group and 2.5 μg/g ± 0.6 μg/g in the Sham-CSO group, with no significant difference between the groups. The content of Evans blue was 19.8 μg/g ± 1.1 μg/g in the MCAO-Con group (^##^*p* < 0.01 vs. Sham-Con group), and CSO treatment significantly decreased the content of Evans blue to 13.9 μg/g ± 2.8 μg/g (^*^p < 0.05 vs. MCAO-Con group).

**Fig. 4:**
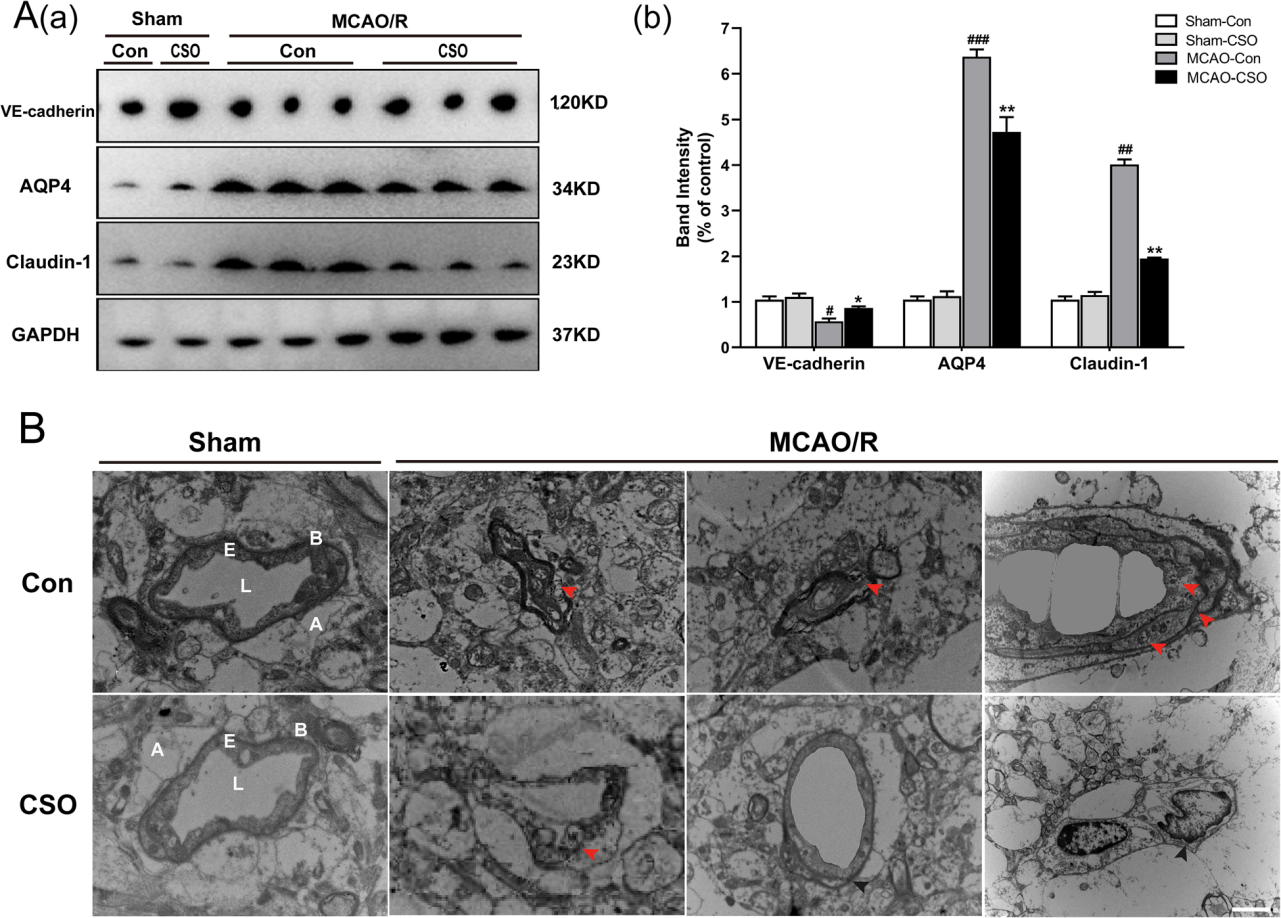
CSO treatment relieved the physiological conditions of BBB in the ischemic penumbra. **A** CSO pretreatment increases VE-cadherin protein and reduces AQP4 and Claudin-1 protein expression in the ischemic penumbra 24 h after reperfusion. **A** **(a)** Cropped gels and blots showing the protein expression of the VE-cadherin, AQP4, and Claudin-1. **A(b)** Graph of the protein expression of the VE-cadherin, AQP4, and Claudin-1. ^###^*p* < 0.001, ^##^*p* < 0.01, ^#^*p* < 0.05 vs. Sham-Con group, ^**^p < 0.01, ^*^p < 0.05 vs. MCAO-Con group. *n* = 6 per group. **B** Representative transmission electron micrographs showing the high-magnification images of ultrastructural changes in different groups of rats. Red arrowheads indicate disruption of the plasma membrane, basal membrane, gap junctions, organelles, accumulation of glycogen, and swollen endothelial cytoplasm. Black arrowheads indicate intact vascular ultrastructure. A: astrocyte end-feet; B: basal lamina; E: endothelial cell; L: lumen. Scale bar = 2 μm. *n* = 3 per group

We next evaluated cerebral edema markers in the ischemia penumbra 24 h after reperfusion. In particular, we focused on VE-cadherin, an important component of the BBB, and AQP4 and Claudin-1, which are strongly expressed by leaky brain microvessels. As shown in Fig. 4A (a, b), western blot analysis indicated no significant differences in the expression of VE-cadherin, AQP4, or Claudin-1 between the Sham-Con and Sham-CSO groups. Twenty-four hours after reperfusion, the MCAO-Con group had significantly decreased expression of VE-cadherin and increased expression of AQP4 and Claudin-1 (^###^*p* < 0.001, ^##^*p* < 0.01, ^#^*p* < 0.05 vs. Sham-Con group). However, compared with the MCAO-Con group, the MCAO-CSO group had significantly increased expression of VE-cadherin and decreased expression of AQP4 and Claudin-1(^**^p < 0.01, ^*^p < 0.05).

BBB disruption is the main pathological mechanism leading to ischemic brain edema, so we used transmission electron microscopy (TEM) to measure the BBB ultrastructure in the four groups of rats. As shown in Fig. 4B, no obvious difference in the BBB ultrastructure was observed between the Sham-Con and Sham-CSO groups under physiological conditions. However, striking effects emerged in the MCAO groups 24 h after reperfusion. Swollen astrocyte end-feet were clearly evident in the ischemic penumbra of the MCAO-Con and the MCAO-CSO groups, and the effect was exacerbated in the MCAO-Con group, which exhibited elevated astrocyte swelling, discontinuous plasma membranes, disturbed gap junctions, broken organelles, swollen endothelial cytoplasm, and rough basal membranes. Finally, pretreatment with CSO significantly reduced the ultrastructure damage in MCAO-CSO group observed with TEM.

### CSO treatment significantly inhibited TLR4/NF-κB mediated activation of microglia and astrocytes in the ischemia penumbra

**Fig. 5:**
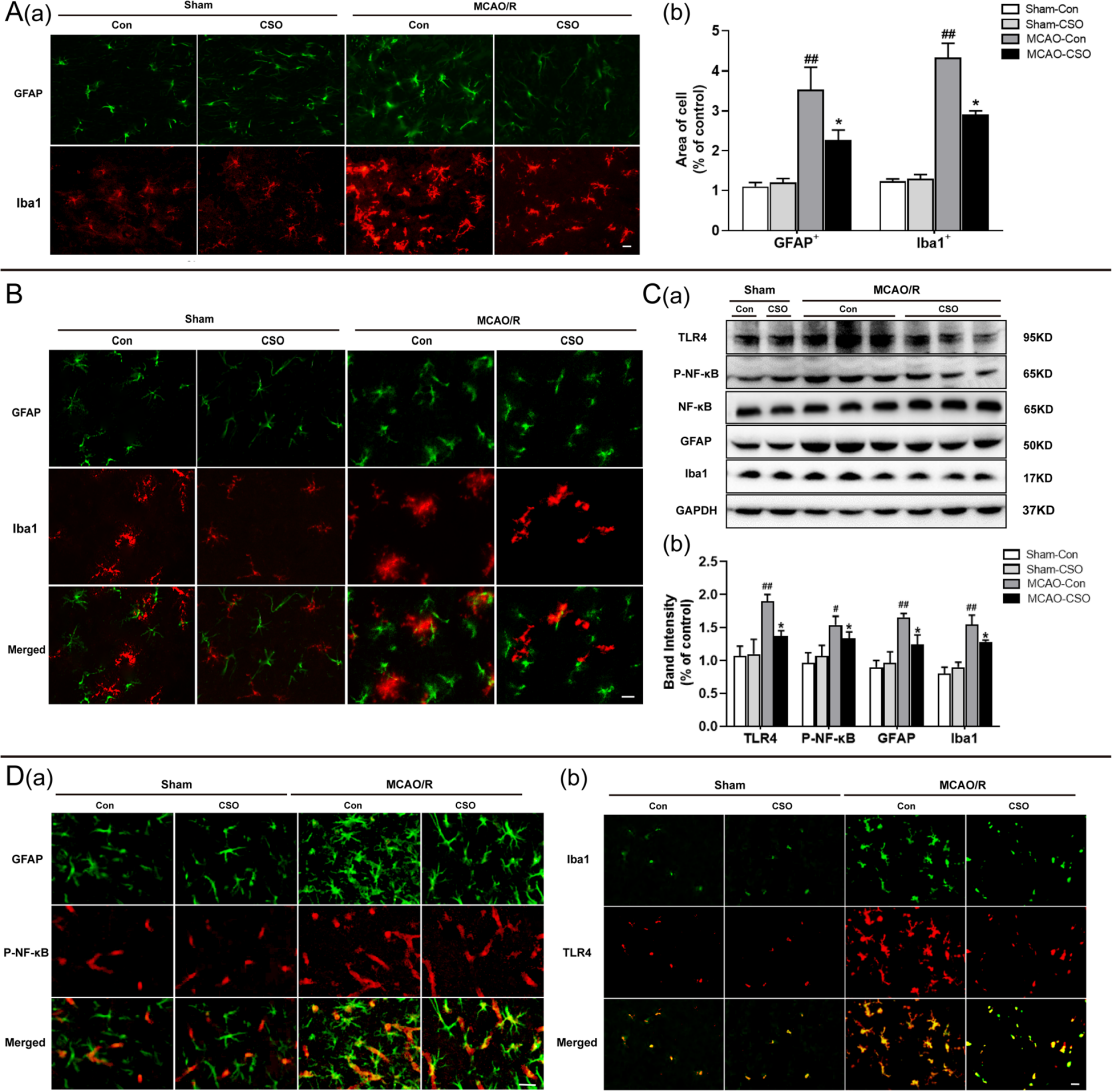
CSO treatment relieved TLR4/NF-κB-mediated hyperactivation of astrocyte and microglia in the ischemic penumbra. **A** CSO treatment relieved astrocyte and microglia activation, respectively. **A**(**a**) Representative immunofluorescence images showing the morphologies of the astrocyte labeled with GFAP and microglia labeled with Iba1 in the ischemic penumbra 24 h after reperfusion. Scale bar = 20 μm. **A**(**b**) The average area of single microglial cells labeled by Iba1 and single astrocytic cells labeled by GFAP in the ischemic penumbra. ^##^*p* < 0.01 vs. Sham-Con group, ^*^p < 0.05 vs. MCAO-Con group. *n* = 5 per group. **B** CSO treatment relieved astrocyte and microglia co-activation. Representative immunofluorescence images showing the morphologies of the astrocyte labeled with GFAP and microglia labeled with Iba1 in the ischemic penumbra under normal conditions, and the activated microglia was close to the activated astrocyte in the ischemic penumbra 24 h after reperfusion, and CSO significantly inhibited co-activation of microglia and astrocyte. Scale bar = 20 μm. **C** CSO treatment reduced the expression of TLR4, P-NF-κB, GFAP, and Iba1 in the ischemic penumbra 24 h after reperfusion. **C**(**a**) Cropped gels and blots showing the protein expression of the TLR4, P-NF-κB, NF-κB, GFAP, and Iba1 in the ischemic penumbra 24 h after reperfusion. **C(b)** Graph of the protein expression of the TLR4, P-NF-κB, GFAP, and Iba1 in the ischemic penumbra 24 h after reperfusion. ^##^*p* < 0.01, ^#^*p* < 0.05 vs. Sham-Con group, ^*^p < 0.05 vs. MCAO-Con group. *n* = 6 per group. **D** CSO treatment relieved P-NF-κB-mediated astrocyte activation and TLR4-mediated microglia activation, respectively. **D**(**a**) Representative immunofluorescence images showing the colocalization of P-NF-κB and GFAP in the ischemic penumbra 24 h after reperfusion. Scale bar = 5 μm. **D**(**b**) Representative immunofluorescence images showing the colocalization of TLR4 and Iba1 in the ischemic penumbra 24 h after reperfusion. Scale bar = 20 μm

In the next set of analyses, we measured microglial and astrocytic activation in the ischemia penumbra across the four groups. As shown in Fig. 5A (a, b), there were no obvious differences in microglial and astrocytic activation between the Sham-Con and Sham-CSO groups under physiological conditions. Compared with the Sham-Con group, the astrocytes (GFAP-positive) and microglia (Iba1-posittive) in the MCAO-Con group were more activated (^##^*p* < 0.01), as evident from large soma and short, coarse cytoplasmic and hypertrophic processes. However, CSO treatment significantly attenuated the microglial and astrocytic activation induced by MCAO-R injury (^*^p < 0.05 vs. MCAO-Con group). Additionally, we found that the activated microglia were close to the activated astrocytes in the ischemia penumbra in both the MCAO-Con and MCAO-CSO groups, indicating possible co-activation of microglia and astrocytes following ischemic stroke. In keeping with this conclusion, CSO treatment significantly inhibited co-activation of microglia and astrocytes in the MCA-CSO group compared with the MCAO-Con group (Fig. 5B).

In addition, we used western blot 24 h after reperfusion to measure the protein expression of Iba1 and GFAP in the ischemia penumbra, which reflects the activation of microglia and astrocytes, respectively. As shown in Fig. 5C (a, b), there was no significant difference in expression of Iba1 or GFAP between the Sham-Con and Sham-CSO groups. The expression of Iba1 and GFAP in the MCAO-Con group was significantly higher than in the Sham-Con group (^##^*p* < 0.01). Remarkably, CSO treatment significantly decreased the expression of Iba1 and GFAP compared with the MCAO-Con group (^*^p < 0.05).

Next, in order to further investigate whether microglia and astrocyte activation are mediated by TLR4 and NF-κB, we measured TLR4, phospho-NF-κB (P-NF-κB), and NF-κB expression levels in the ischemia penumbra. As shown in Fig. 5C (a, b), no significant difference in TLR4 and P-NF-κB expression was found between the Sham-Con and Sham-CSO groups. TLR4 and P-NF-κB protein levels were significantly increased in the MCAO-Con group (^##^*p* < 0.01, ^#^*p* < 0.05 vs. Sham-Con group), and CSO significantly inhibited the expression of TLR4 and P-NF-κB compared with the MCAO-Con group (^*^p < 0.05).

Finally, to verify that the activation of microglia and astrocytes were initiated by TLR4 and NF-κB respectively, we investigated the colocalization of GFAP and P-NF-κB and the colocalization of Iba1 and TLR4 in the ischemia penumbra. As shown in Fig. 5D (a), P-NF-κB was predominantly expressed by astrocytes, and there were no significant differences in the expression of GFAP or P-NF-κB between the Sham-Con and Sham-CSO groups. The expression of GFAP and P-NF-κB in the MCAO-Con group was significantly higher than in the Sham-Con group, and CSO treatment significantly inhibited the expression of GFAP and P-NF-κB compared with the MCAO-Con group. Similarly, as shown in Fig. 5D (b), TLR4 was predominantly expressed by the microglia and there was no significant difference in the expression of Iba1 and TLR4 between the Sham-Con and Sham-CSO groups. Iba1 and TLR4 levels were significantly higher in the MCAO-Con group, and CSO treatment significantly inhibited the expression of Iba1 and TLR4 compared with the MCAO-Con group.

### CSO treatment significantly inhibited inflammation in the ischemia penumbra

**Fig. 6:**
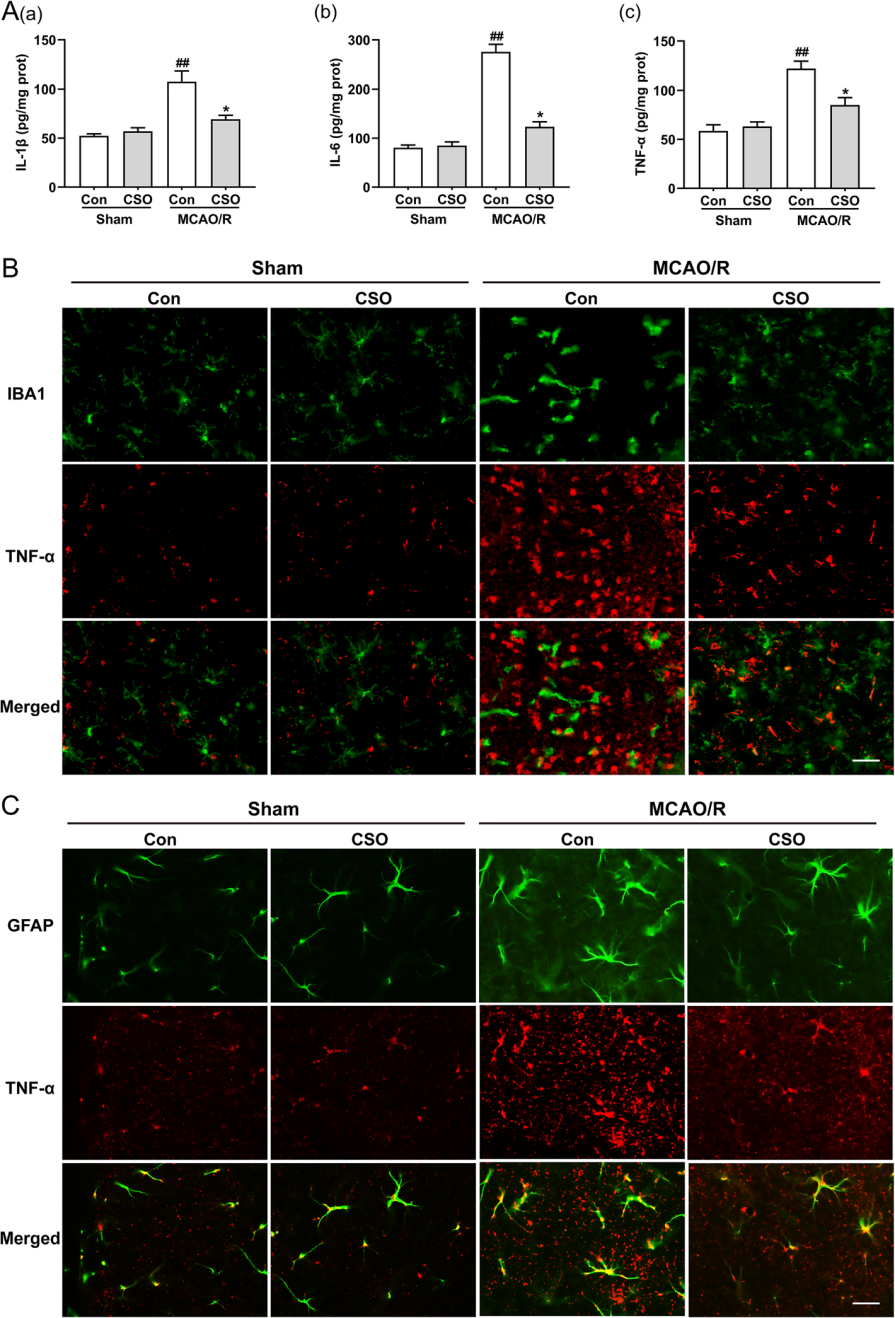
CSO treatment attenuated neuroinflammation in the ischemic penumbra. **A** CSO attenuated the accumulation of pro-inflammatory cytokines in the ischemic penumbra. Levels of IL-1β (a), IL-6 (b), and TNF-α (c) in the ischemic penumbra 24 h after reperfusion. ^##^*p* < 0.01 vs. Sham-Con group, ^*^p < 0.05 vs. MCAO-Con group. *n* = 5 per group. **B** Localization and expression of Iba1 and TNF-α in the ischemic penumbra after MCAO/R. Iba1 and TNF-α partial colocalized in the ischemic penumbra under normal conditions, and their expression increased after reperfusion (24 h), and their expression decreased after treatment with CSO. Scale bar = 10 μm. *n* = 5 per group. **C** Localization and expression of GFAP and TNF-α in the ischemic penumbra after MCAO/R. GFAP and TNF-α partially colocalized in the ischemic penumbra under normal conditions, and their expression increased after reperfusion (24 h), and their expression decreased after treatment with CSO. Scale bar = 10 μm. *n* = 5 per group

We measured the expression of proinflammatory cytokines (IL-1β, IL-6, and TNF-α) in the ischemia penumbra 24 h after reperfusion. As shown in Fig. 6A (a), the level of IL-1β was 52.3 pg/mg protein ± 2.1 pg/mg protein in the Sham-Con group and 57.0 pg/mg protein ± 3.6 pg/mg protein in the Sham-CSO group, with no significant difference between the groups. The level of IL-1β was 107.3 pg/mg protein ± 11.0 pg/mg protein in the MCAO-Con group (^##^*p* < 0.01 vs. Sham-Con group), and CSO treatment significantly decreased the level of IL-1β to 69.3 pg/mg protein ± 4.0 pg/mg protein (^*^p < 0.05 vs. MCAO-Con group). As shown in Fig. 6A (b), the level of IL-6 was 80.3 pg/mg protein ± 5.5 pg/mg protein in the Sham-Con group and 85.0 pg/mg protein ± 7.2 pg/mg protein in the Sham-CSO group, with no significant difference between the groups. The level of IL-6 was 275.7 pg/mg protein ± 15.3 pg/mg protein in the MCAO-Con group (^##^p < 0.01 vs. Sham-Con group), and CSO treatment significantly decreased the level of IL-6 to 123.3 pg/mg protein ± 10.1 pg/mg protein (^*^p < 0.05 vs. MCAO-Con group). As shown in Fig. 6A (c), the level of TNF-α was 58.7 pg/mg protein ± 6.2 pg/mg protein in the Sham-Con group and 63.3 pg/mg protein ± 4.6 pg/mg protein in the Sham-CSO group, with no significant difference between the groups. The level of TNF-α was 121.9 pg/mg protein ± 7.7 pg/mg protein in the MCAO-Con group (^##^*p* < 0.01 vs. Sham-Con group), and CSO treatment significantly decreased the level of TNF-α to 85.0 pg/mg protein ± 7.6 pg/mg protein (^*^p < 0.05 vs. MCAO-Con group).

In order to provide additional evidence of the effect of CSO on inflammation following ischemic stroke, we used immunofluorescence staining to detect the expression of the microglia marker Iba1 and pro-inflammatory factor TNF-α in the ischemia penumbra 24 h after reperfusion. As shown in Fig. 6B, there was no significant difference in the expression level of Iba1 and TNF-α between the Sham-Con and Sham-CSO groups. The expression of Iba1 and TNF-α was significantly higher in the MCAO-Con group compared with the Sham-Con group. Additionally, the levels of Iba1 and TNF-α in the MCAO-CSO group were markedly decreased compared to the MCAO-Con group. We also measured the expression of the astrocyte marker GFAP and pro-inflammatory factor TNF-α in the ischemia penumbra 24 h after reperfusion. As shown in Fig. 6C, no significant difference in the expression of GFAP and TNF-α was found between the Sham-Con and Sham-CSO groups. The expression of GFAP and TNF-α significantly increased in the MCAO-Con group compared to the Sham-Con group. Finally, rats in the MCAO-CSO group had significantly lower levels of GFAP and TNF-α than those in the MCAO-Con group.

### CSO treatment inhibited A1 type reactive astrocytes and promoted A2 type reactive astrocytes in the ischemia penumbra

**Fig. 7:**
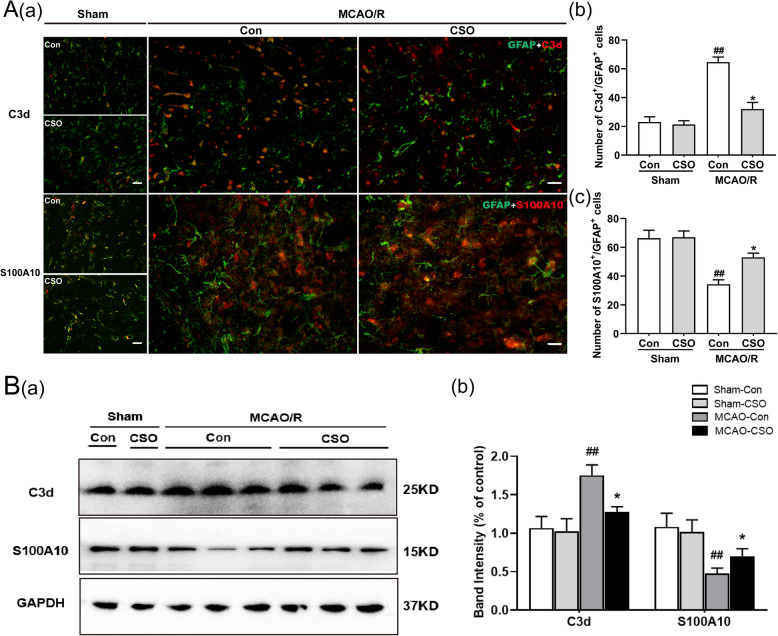
CSO treatment affected the activation of A1/A2 type reactive astrocytes in the ischemic penumbra. **A** CSO treatment decreased the number of A1 type astrocytes and increased the number of A2 type astrocytes. **A**(**a**) Representative immunofluorescence images showing co-localization of the astrocyte labeled with GFAP and A1 astrocyte labeled with C3d, and co-localization of the astrocyte labeled with GFAP and A2 astrocyte labeled with S100A10 in the ischemic penumbra 24 h after reperfusion. Scale bar in the Sham-Con and Sham-CSO groups = 50 μm. Scale bar in the MCAO-Con and MCAO-CSO groups = 20 μm. A(**b**) Percentage of C3d-positive cells that are GFAP-positive in the ischemic penumbra. ^##^*p* < 0.01 vs. Sham-Con group, ^*^p < 0.05 vs. MCAO-Con group. *n* = 5 per group. (c) Percentage of S100A10-positive cells that are GFAP-positive in the ischemic penumbra. ^##^*p* < 0.01 vs. Sham-Con group, ^*^p < 0.05 vs. MCAO-Con group. *n* = 5 per group. **B** CSO treatment decreased C3d protein and increased S100A10 protein expression in the ischemic penumbra 24 h after reperfusion. **B**(**a**) Cropped gels and blots showing the protein expression of the C3d and S100A10. **B**(**b**) Graph of the protein expression of the C3d and S100A10. ^##^*p* < 0.01 vs. Sham-Con group, ^*^p < 0.05 vs. MCAO-Con group. *n* = 6 per group

We investigated the presence of A1 type astrocytes (labeled by C3d/GFAP) and A2 type astrocytes (labeled by S100A10/GFAP) in the ischemia penumbra across the four groups, and subsequently assessed the influence of CSO treatment on the number of peri-infarct C3d/GFAP-positive cells and S100A10/GFAP-positive cells by performing immunofluorescence staining. As shown in Fig. 7A (a, b, c), there was no significant difference in the number of C3d/GFAP-positive cells and S100A10/GFAP-positive cells between the Sham-Con and Sham-CSO groups. Additionally, C3d/GFAP-positive cells were significantly increased and S100A10/GFAP-positive cells were significantly decreased in MCAO-Con group compared with the Sham-Con group (^##^*p* < 0.01). The CSO-treated MCAO rats had significantly fewer C3d/GFAP-positive cells than did the rats in the MCAO-Con group (^*^p < 0.05), and the number of S100A10/GFAP-positive cells in the MCAO-CSO group was significantly greater than in the MCAO-Con group (^*^p < 0.05).

Finally, we used western blot analysis to measure the protein expression of the A1 type astrocyte marker C3d and the protein expression of the A2 type astrocyte marker S100A10 in the ischemia penumbra. As shown in Fig. 7B (a, b), there were no significant differences in C3d or S100A10 expression between the Sham-Con and Sham-CSO groups. The C3d protein level was markedly increased in the MCAO-Con group (^##^*p* < 0.01 vs. Sham-Con group) and this effect was reduced by CSO treatment (^*^p < 0.05). The S100A10 protein level was markedly decreased in the MCAO-Con group (^##^*p* < 0.01 vs. Sham-Con group), and this effect was reversed by CSO treatment (^*^p < 0.05).

## Discussion

Most of the scientific studies involving CSO use it as the solvent for lipid-soluble drugs such as estrogen and ketorolac [5, 6]. An influential animal study published in *Science* found that in dogs, thrombosis and thromboembolic disease associated with atherosclerosis occurred with diets containing beef tallow and lard or coconut oil but not with diets using CSO as a source of fat. These results supported the conclusion that CSO may provide the basis for therapeutic intervention strategies against thromboembolic diseases [9]. However, the effect of CSO on ischemic stroke has not been explored before. In this study, we found that CSO treatment significantly improved neurological deficits, reduced infarction volume, and increased neuronal survival in the ischemic penumbra, suggesting that CSO treatment could be a new strategy for preventing ischemic stroke. It is worth noting that CSO as a drug solvent is commonly administered by subcutaneous injection [5, 6], so we employed subcutaneous injection in the current study. Additionally, CSO is edible but it is not clear whether edible CSO is neuroprotective, which is a question that should be addressed in future research.

Several studies have shown that due to its anti-inflammatory effects, CSO provides a wide array of health benefits including protection against peripheral tissue injury [7, 8]. For example, one recent study found that CSO treatment protected against inflammatory bowel disease (IBD) by reducing the expression of inflammatory cytokines such as TNF-α, IL-1β, IL-6, and IL-17 [7]. Another study reported that ketorolac in saline produced 8 h of effective antinociceptive and anti-inflammatory effects, whereas ketorolac in CSO vehicles produced 78 h of antinociceptive and anti-inflammatory effects [5]. While these results are promising, previous research has not specifically examined the effects of CSO on the neuroinflammation induced by ischemic stroke injury.

Neuroinflammation induced by activated microglia and astrocytes contributes substantially to ischemic stroke injury [10–12]. Immediately after ischemic stroke, microglia and astrocytes are excessively activated, as evident from large cell bodies and hypertrophic processes, and they release robust levels of inflammatory cytokines, such as IL-1, IL-6, and TNF-α, which contribute to severe inflammatory responses and aggravate brain injury [10–12]. Inhibiting the activation of microglia and astrocytes during ischemic stroke could significantly alleviate this brain injury [24]. In the current study, we evaluated the effect of CSO on the neuroinflammatory processes following ischemic stroke injury which are mediated by microglia and astrocyte activation. We found that CSO treatment significantly inhibited the hyperactivation of microglia and astrocytes and significantly decreased the expression levels of proinflammatory cytokines (IL-1β, IL-6, and TNF-α) in the ischemia penumbra after MCAO-R injury. However, the underlying mechanism remains unclear.

TLR4 is primarily expressed in microglia and mediates microglial activation and inflammatory response [25]. In addition, the transcription factor NF-κB plays a pivotal role in initiating neuroinflammation following ischemic stroke; activating NF-κB significantly promotes the expression and secretion of inflammation-related genes in astrocytes [26]. In the current study, we found that following MCAO-R injury, the activation of microglia and astrocytes were initiated through TLR4 and NF-κB, respectively. Additionally, CSO treatment significantly inhibited TLR4 and NF-κB expression in the ischemia penumbra. Our previous studies found that inhibiting the expression of TLR4 and NF-κB significantly alleviates cerebral ischemia injury [15, 27], and another recent study verified that suppression of the TLR4/NF-κB signaling pathway improved cerebral ischemia-reperfusion injury in rats [28]. Thus, we demonstrated that CSO treatment significantly inhibits TLR4/NF-κB mediated activation of microglia and astrocytes and the downstream inflammatory response. However, further research is needed to specify the receptors and signaling pathways involved in CSO’s regulation of TLR4 and NF-κB.

**Fig. 8:**
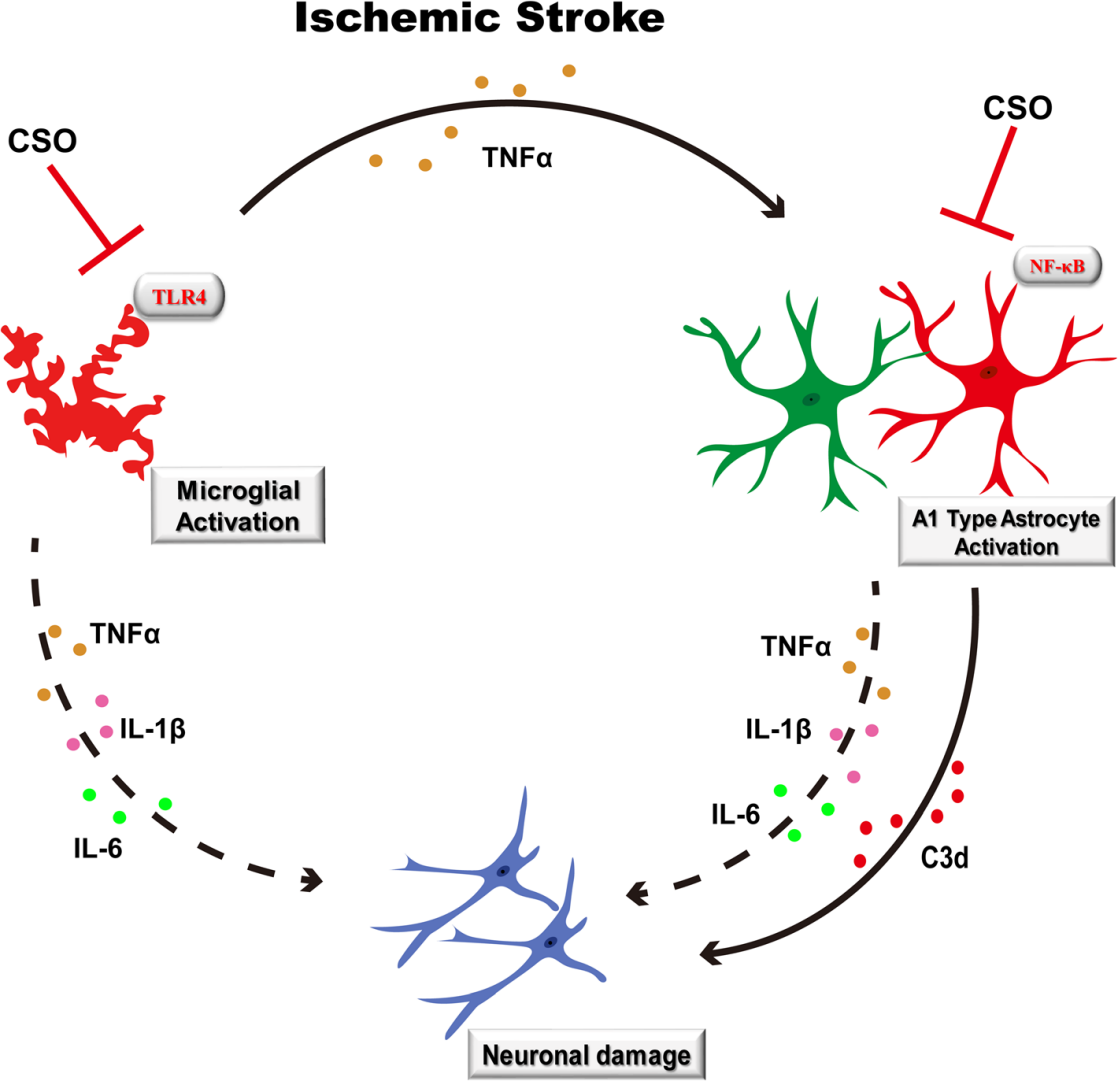
A scheme of regulation of ischemic stroke injury by CSO treatment. Immediately after ischemic stroke, microglia and astrocytes were excessively activated and released robust inflammatory cytokines, such as IL-1, IL-6, and TNF-α, which contributed to severe inflammatory response and thus aggravated brain injury. And CSO treatment could reduce the expression and release of proinflammatory cytokines (IL-1β, IL-6, and TNF-α) by inhibiting microglia and astrocyte activation induced by ischemic stroke injury. In addition, ischemic stroke injury leads to TLR4-mediated microglia activation with the release of TNF-α, which induces NF-κB-mediated A1 astrocyte activation and the release of neurotoxic complement C3d directly leading to neuron death; this pathway was alleviated by CSO treatment

Previous research has reported evidence of crosstalk between astrocytes and microglia, which may contribute to the neuroinflammation that ultimately induces postoperative cognitive dysfunction (POCD) [29]. In LPS-treated co-cultures of astrocytes and microglia, microglia P2Y_6_ receptors induce the release of nitric oxide, which causes astrocyte apoptosis [30]. In the current study, we found that some of the activated microglia and astrocytes were adjacent to each other in the penumbra, indicating potential “co-activation” and interaction between activated microglia and astrocytes. We also found that CSO treatment markedly reduced this co-activation. Recent studies indicate that ischemic stroke induces two types of astrocyte activation. One is the neurotoxic A1 type, which is induced by activated microglia that secrete IL-1α, TNF-α, and C1q; A1 type astrocytes can release neurotoxic complement C3d, leading to neuronal death. The second type is the neuroprotective A2 type, which promotes neuronal survival and tissue repair [17, 18]. We found that CSO treatment significantly decreased A1 type astrocyte activation (as evident from the downregulation of C3d expression) and increased A2 type astrocyte activation. Additionally, we identified NF-κB as the key molecule that mediates A1 type astrocyte activation [17]. Taken together, these findings indicate that CSO treatment inhibited TLR4-mediated microglial activation and TNF-α secretion, thereby inhibiting NF-κB-mediated A1 astrocyte activation and reducing C3d expression and secretion, ultimately alleviating neuronal damage induced by ischemic stroke injury (Fig. 8).

Brain edema is a life-threatening consequence of stroke, potentially leading to brain herniation and death [31]. BBB integrity is affected by astrocytes and endothelial cell and its junctions including adherens junctions, VE-cadherin and tight junctions, claudin-5, and claudin-1 [32–34]. Endothelial cells express VE-cadherin, which promotes junction stability through their interaction with the actin cytoskeleton [32]. Claudin-1 is rarely expressed in the normal BBB but is strongly expressed in leaky brain microvessels after stroke [33]. Additionally, AQP4 was found at the foot processes of the astrocytes which mediate water accumulation in the cytotoxic edema associated with the onset of brain stroke [34]. We found that CSO treatment alleviated brain edema by protecting the BBB integrity, increasing VE-cadherin protein expression, and reducing claudin-1 and AQP4 protein expression. Activated microglia and astrocytes increase neuroinflammation which leads to the disruption of the BBB [12], and our results indicate that CSO alleviates BBB damage and brain edema induced by ischemic stroke by inhibiting inflammation. These promising results suggest the need for further research into the specific mechanisms underpinning CSO’s BBB-protective effects.

## Conclusion

In conclusion, the current findings indicate that CSO treatment can alleviate ischemic stroke injury via reducing inflammatory microglia and astrocytic activation, which was correlated to the inhibition of TLR4/NF-κB pathway and the reduction of A1 type neurotoxic astrocyte activation. Our results suggest that CSO treatment is an attractive strategy for prevention of ischemic stroke.

## Supplementary information

**Additional file 1: Supplementary Figure 1.** The regional cerebral blood flow monitoring conducted along the MCAO/R surgeries.

## Data Availability

The datasets during and/or analyzed during the current study are available from the corresponding author on reasonable request.

## Abbreviations

BBB: Blood–brain barrier
CNS: Central nervous system
CSO: Cottonseed oil
GFAP: Glial fibrillary acidic protein
HE: Hematoxylin and eosin
IBD: Inflammatory bowel disease
IL-1β: Interleukin-1β
IL-6: Interleukin-6
MCAO-R: Middle cerebral artery occlusion and reperfusion
PBS: Phosphate-buffered solution
POCD: Postoperative cognitive dysfunction
SD: Standard deviation
TLR4: Toll-like receptor 4
NF-κB: Nuclear factor kappa B
TEM: Transmission electron microscopy
TTC: 2,3,5-triphenyltetrazolium chloride
TNF-α: Tumor necrosis factor α
