# Supplementary material for: Cottonseed oil alleviates ischemic stroke injury by inhibiting the inflammatory activation of microglia and astrocyte
Source: J Neuroinflammation. 2020 Sep 11;17:270. doi: 10.1186/s12974-020-01946-7 (PMC7488511; doi:10.1186/s12974-020-01946-7)
Supplement: Supplementary file 1 — Additional file 1: Supplementary Figure 1. The regional cerebral blood flow monitoring conducted along the MCAO/R surgeries. [file 12974_2020_1946_MOESM1_ESM.docx]

**Supplementary Figure 1**


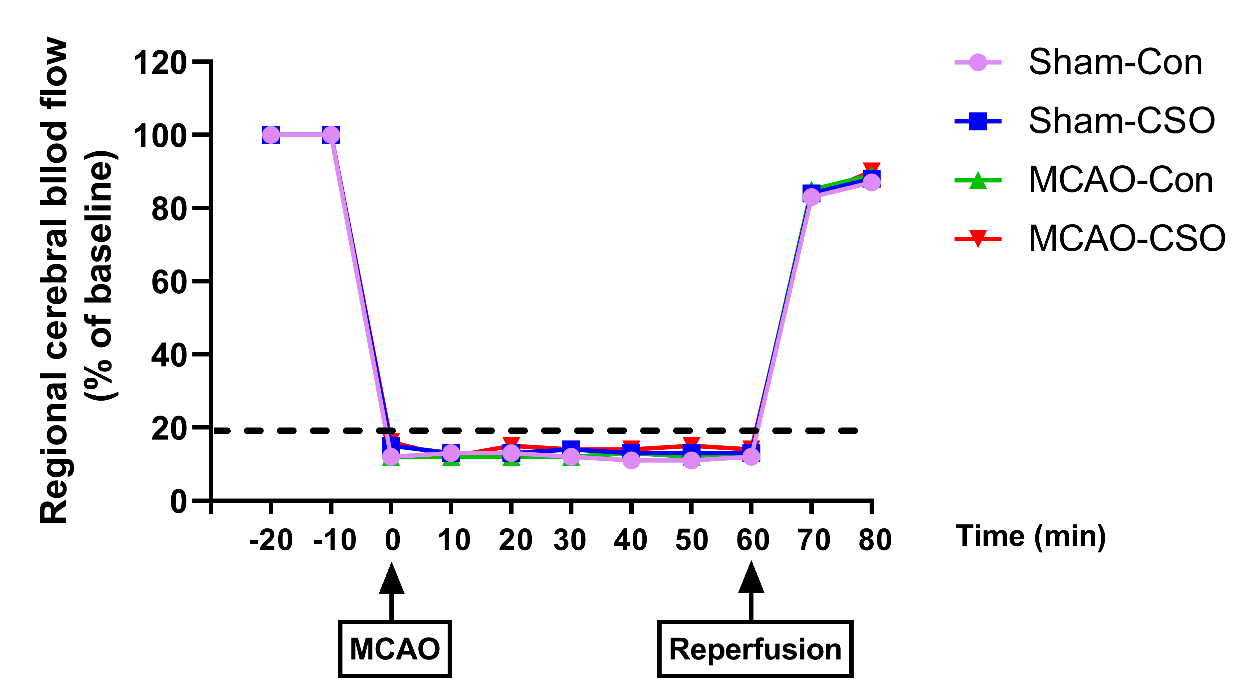


**Supplementary Figure 1. The regional cerebral blood flow monitoring conducted along the MCAO/R surgeries.**
